# Partial substitution of chemical fertilizer by *Trichoderma* biofertilizer improved nitrogen use efficiency in wolfberry (*Lycium chinense*) in coastal saline land

**DOI:** 10.3389/fpls.2023.1225028

**Published:** 2023-10-09

**Authors:** Kun Yan, Huimin Mei, Yanan Ruan, Shunyang Yu, Hongyan Su, Yibo Zhi, Suxin Li, Yanan Sun

**Affiliations:** ^1^ School of Agriculture, Ludong University, Yantai, China; ^2^ School of Life Sciences, Liaoning University, Shenyang, China; ^3^ CAS Key Laboratory of Coastal Environmental Processes and Ecological Remediation, Yantai Institute of Coastal Zone Research, Chinese Academy of Sciences (CAS), Yantai, China

**Keywords:** nitrogen, photosynthesis, stable isotopic compositions, *Trichoderma*, wolfberry

## Abstract

A two-year field trial was conducted to investigate the effects of partial substitution of chemical fertilizer (CF) by *Trichoderma* biofertilizer (TF) on nitrogen (N) use efficiency and associated mechanisms in wolfberry (*Lycium chinense*) in coastal saline land. As with plant biomass and fruit yield, apparent N use efficiency and plant N accumulation were also higher with TF plus 75% CF than 100% CF, indicating that TF substitution promoted plant growth and N uptake. As a reason, TF substitution stabilized soil N supply by mitigating steep deceases in soil NH_4_^+^-N and NO_3_^–^N concentrations in the second half of growing seasons. TF substitution also increased carbon (C) fixation according to higher photosynthetic rate (Pn) and stable ^13^C abundance with TF plus 75% CF than 100% CF. Importantly, leaf N accumulation significantly and positively related with Pn, biomass, and fruit yield, and structural equation modeling also confirmed the importance of the causal relation of N accumulation coupled with C fixation for biomass and yield formation. Consequently, physiological and agronomical N use efficiencies were significantly higher with TF plus 75% CF than 100% CF. Overall, partial substitution of CF by TF improved N use efficiency in wolfberry in coastal saline land by stabilizing soil N supply and coupling N accumulation with C fixation.

## 1 Introduction

Saline land is a worldwide resource that must be used rationally to satisfy increasing food demand (Rozema and Schat, 2013; Qin et al., 2015; Nikalje et al., 2017; Zhang et al., 2021). In China, the Yellow River Delta has a large area of coastal saline land with poor agricultural productivity. An important barrier to increase productivity is that nitrogen (N) fertilizer is not used efficiently in saline cropland, because salinity stress inhibits plant N uptake and assimilation (Ashraf et al., 2018). Nitrogen is also easily lost in saline soil, further restricting plant N uptake (Duan et al., 2018; Zhu et al., 2020; Li et al., 2020b). In the Yellow River Delta, hydrological and climatic characteristics such as shallow groundwater table, concentrated rainfall, and high evaporation to precipitation ratio aggravate soil N loss in nitrate leaching and NH_3_ volatilization (Zhu et al., 2021).

Nitrogen is an essential macronutrient for plants, and crop N deficiency under saline stress is typically expressed in poor growth and yield (Abouelsaad et al., 2016; Yu et al., 2016; Cui et al., 2019). Plant N use efficiency is a comprehensive proxy of N uptake and use and is closely associated with photosynthetic carbon (C) fixation (Wu et al., 2019). Photosynthesis provides the C skeleton and energy for N uptake and assimilation, whereas N uptake and assimilation contribute to the synthesis of photosynthetic pigments and C assimilation enzymes. Thus, coordination between C and N processes is responsible for efficient N use and healthy plant growth. In addition to the decrease in N use, saline stress also depresses photosynthesis by inducing leaf stomatal closure and photosystem photoinhibition (Yan et al., 2013; Yan et al., 2020; Zhao et al., 2020). However, whereas many studies focus on the response of either photosynthesis or N accumulation to salt stress, only a few simultaneously examine salt-induced variations in both processes by using pot experiments (Nazar et al., 2011; Liu et al., 2013). Thus, to determine plant N use in saline land, the synergism between photosynthesis and N accumulation needs to be explored further by field trials.

Chemical fertilizer is habitually overused to increase crop yields in China, but the yield-increasing effect has reached a bottleneck, and much chemical N is wasted to exacerbate the environmental burden (Liang et al., 2019; Li et al., 2021). Partial organic substitution of chemical fertilizer is a sustainable measure that has been used to solve the problem in various agricultural ecosystems, including tea plantations, vegetables, and field crops, because it can guarantee yields while simultaneously reduce chemical N input and loss (Tang et al., 2019; Zhou et al., 2019; Ji et al., 2020; Li et al., 2021; Hou et al., 2023). Soil organic matter is generally low in saline land, which can decrease microbial N immobilization and result in large N loss, and accordingly, organic amendment is a feasible approach to mitigate N loss and improve crop yield by increasing N retention in saline soil (Xiao et al., 2020; Zhu et al., 2020). Plant growth-promoting microorganisms have recently gained increasing attention, because they can be used in a green approach to abate saline barriers, in contrast to other remediation protocols with high investment costs (Arora et al., 2020; Kaushal, 2020). Biofertilizer is a type of organic fertilizer rich in plant growth-promoting microorganisms, and compared with organic fertilizer, biofertilizer application combined with chemical N more effectively reduces N loss in saline soil and increases crop yield (Wang et al., 2018; Xue et al., 2021). *Trichoderma* is a growth-promoting fungus and also can strengthen plant resistance to salt stress by regulating hormone synthesis, increasing osmotic and antioxidant defenses, and maintaining ion balance and photosynthetic performance (Chen et al., 2016; Zhang et al., 2016; Zhang et al., 2019a; Zhang et al., 2019b; Oljira et al., 2020). *Trichoderma* inoculation was evidenced to aid in elevating yield and quality of onion and leafy vegetables iceberg lettuce and rocket and reducing fertilizer investment (Ortega-Garcia et al., 2015; Fiorentino et al., 2018). In addition, it has been well documented that partial substitution of chemical fertilizer by *Trichoderma* biofertilizer (TF) was superior to substitution by organic fertilizer, and could increase soil fertility and nutrient availability, regulate soil microflora composition and enhance crop yield (Cai et al., 2015; Pang et al., 2017; Zhang et al., 2018; Qiao et al., 2019; Liu et al., 2020; Ye et al., 2020; Liu et al., 2021). However, the knowledge about TF application for crop cultivation in saline soil remains very limited and mainly originates from pot experiments. Wang et al. (2018) reported that combined application of TF with urea reduced NH_3_ loss from saline soil and promoted potted sweet sorghum growth, and likewise, TF effectively altered saline soil properties and microbial composition, leading to the increase in potted *Medicago sativa* biomass (Zhang et al., 2020). However, the application of *Trichoderma* spore powder did not improve potted tomato yield under saline irrigation (Daliakopoulosa et al., 2019). Whether TF application can promote crop growth by improving N utilization in coastal saline land still needs to be examined by field trials. In particular, it is largely unknown whether TF substitution can increase N retention in saline soil and stabilize soil N supply.

Wolfberry (*Lycium chinense*) is a deciduous shrub in the Solanaceae, and its fruit has long been used as traditional medicine and health food in China. Wolfberry may be an ideal economic crop to plant in coastal saline land, because it is a halophyte naturally distributed in coastal zones. However, despite high adaptability to salt, saline stress also inhibits wolfberry photosynthesis and growth, and cultivation methods need to be developed to achieve high fruit yields in saline land (Feng et al., 2017; Dimitrova et al., 2019). Chemical fertilizer was commonly used to improve growth and yield of wolfberry, and the importance of application with organic fertilizer has gradually been recognized, however, it remains unknown whether partial substitution of chemical fertilizer by *Trichoderma* biofertilizer is more superior for wolfberry growth in coastal saline land. Recently, a *Trichoderma asperellum* strain was isolated and then successfully prepared as a TF biofertilizer. In this study, the effect of partial substitution of chemical fertilizer (CF) with the TF on N use efficiency in wolfberry was investigated by a two-year field trial in coastal saline land. In particular, mechanisms were examined that affected soil N supply, C and N coupling, and root N absorption. This study may deeply disclose the mechanisms by which TF substitution improve crop growth under salt stress, and also can provide technical guidance for the cultivation of crops in saline land.

## 2 Materials and methods

### 2.1 *Trichoderma* biofertilizer preparation

*Trichoderma asperellum* strain used in the study was deposited in the China general microbiological culture collection center (No. 13187) in Beijing. Apple residue is a typical organic waste in Yantai city, China and has been utilized as the material to prepare TF by us. A *Trichoderma* potato glucose agar plate was punched to obtain three 1 cm diameter blocks, which were shaken in potato glucose liquid medium at 150 rpm and 28°C for 5 d to prepare fermentation broth. Similar with the method in previous studies (Chen et al., 2016; Daneshvara et al., 2023), the fermentation broth was added to apple residue compost and mixed thoroughly for fermentation. After 10 days of fermentation, the product was dried to 30% water content to prepare TF. *Trichoderma* viable colony count was assessed by a dilution method with a selective potato glucose agar plate, which reached 2 × 10^8^ cfu g^-1^ dry weight in the TF. The selective plate contained nystatin, sodium propionate, and streptomycin and was optimized based on Papavizas and Lumsden (1982). In TF, the content of organic matter, total nitrogen, phosphorus pentoxide and potassium oxide was 641.08 mg g^-1^, 26.45 mg g^-1^, 3.37 mg g^-1^ and 27.77 mg g^-1^, respectively.

### 2.2 Field trial

The field trial was conducted in moderate saline land in the Yellow River Delta, Dongying, China (37°17′N, 118°38′E). The site has a warm temperate continental monsoon climate, and annual average temperature and precipitation are approximately 13.5°C and 700 mm, respectively. Soil texture was silty loam, and soil chemical properties were the following: electrical conductivity, 433.7 µs cm^-1^; pH, 8.3; organic matter, 15.3 g kg^-1^; total N, 1.0 g kg^-1^; NH_4_^+^-N, 2.2 mg kg^-1^; NO_3_^–^N, 15.3 mg kg^-1^; Olsen-P, 7.3 mg kg^-1^; and available K, 267.2 mg kg^-1^. The field trial had the following four treatments: (1) NF (no N application); (2) 50% CF plus TF (combined application of 50% chemical N fertilizer and TF); (3) 75% CF plus TF (combined application of 75% CF and TF); (4) 100% CF. In each treatment, four replicate plots (5.5 m × 4 m) were randomly distributed in the field. Plots were separated by 0.3 m wide and 0.3 m high ridges to avoid border effects, and 2 m remained around the plots as an isolation belt. The NF plots were designed as the control without N application. The N application rate was 400 kg ha^-1^ in 100% CF plots, whereas the TF application rate was 5,000 kg ha^-1^ with reduced chemical nitrogen application in 75% CF or 50% CF plus TF treatments. The amount of TF applied was based on a conventional organic fertilizer dose, and the total N application rate in plots with 75% CF plus TF was the same as that in 100% CF plots. Phosphorus application rate was 50 kg ha^-1^ in all plots. Urea and calcium superphosphate were applied as chemical N and P fertilizers, respectively. In April, 2020, twelve two-year bare-rooted wolfberry plants were planted in each plot, and plant and row spacing were 1.5 m. In April, 2020 and 2021, fertilizer was applied to circular furrows, which were 20 cm around plants and 20 cm deep. During the growing seasons in 2020 and 2021, three plants were randomly selected in each plot for measuring gas exchange and modulated and prompt chlorophyll fluorescence parameters. The topsoil (0–20 cm) was also collected in each plot by a five-point sampling method with a soil auger (diameter, 38 mm) in the growing seasons. At the end of October in 2020 and 2021, one plant was selected in each plot and excavated to measure biomass, N and C contents and leaf stable N and C compositions.

### 2.3 Assay of soil nitrogen concentration

Soil samples were sieved through a 2 mm screen and homogenized. One subsample was used to measure soil NH_4_^+^-N and NO_3_^–^N concentrations and moisture. Soil NH_4_^+^-N and NO_3_^–^N were extracted with 2 M KCl for 1 h, and filtrates were analyzed for NH_4_^+^-N and NO_3_^–^N concentrations by a continuous flow analyzer (AutoAnalyzer III, Seal, Germany). Another soil subsample was air-dried, ground, and sieved through a 150 μm screen, and soil total N was determined by an elemental analyzer (VarioMACRO cube, Elementar Analysensysteme, Germany). Soil available K was extracted by using ammonium acetate to measure its content through flame photometry (Lu et al., 2017).

### 2.4 Assay of plant nitrogen content

Plants from plots were separated to leaves, roots, stems, and fruit, which were oven-dried at 60°C to constant weight, ground, and sieved through a 250 μm screen. Powders were digested by H_2_SO_4_–H_2_O_2_, and total N in digests was determined by indophenol blue colorimetry (Ivancic and Degobbis, 1984). In treatments, FDWt, BDWt, and Nt indicate fruit dry weight, plant total biomass, and N accumulation, respectively, whereas in the control, FDWc, BDWc, and Nc respectively indicate those variables. The variable Nf is the amount of N applied. Agronomic, apparent, and physiological N use efficiencies were calculated as (FDWt-FDWc)/Nf, (Nt-Nc)/Nf, and (BDWt-BDWc)/(Nt-Nc), respectively (Chen et al., 2020).

### 2.5 Measurements of leaf stable carbon and nitrogen isotopic compositions and nitrogen and carbon concentrations

Fully expanded leaves in plots were selected, oven-dried at 60°C to constant weight, ground, and sieved through a 250 μm screen. Leaf powder (0.06 mg) was packed in tin capsules, which were sealed and measured using an Elemental Analyzer (FlashEATM 1112, ThermoScientific, Germany) coupled with an isotope ratio mass spectrometer (Finnigan Delta Plus XPTM, ThermoScientific). Stable C and N isotope compositions were expressed using standard delta notation: (δ, ‰) = Rsample/Rstandard - 1, where Rsample and Rstandard were isotopic ratios in samples and standards, respectively. Total N and C in leaf powders were also measured using an elemental analyzer (VarioMACRO cube).

### 2.6 Gas exchange and modulated and prompt chlorophyll fluorescence measurements

In fully expanded leaves in plots, gas exchange and modulated chlorophyll fluorescence parameters were simultaneously detected by using an open photosynthetic system (LI-6400XTR, Li-Cor, USA) equipped with a fluorescence leaf chamber (6400-40 LCF, Li-Cor). Temperature and CO_2_ concentration in the leaf cuvette were set at 25°C and 400 μmol mol^-1^, respectively. Actinic light intensity was set at 1200 µmol m^-2^ s^-1^ in the field trial. Photosynthetic rate (Pn) and stomatal conductance (Gs) were simultaneously recorded, and modulated chlorophyll fluorescence was also recorded to calculate photosynthetic electron transport rate (ETR) according to a previous study (Yan et al., 2020).

A multifunctional plant efficiency analyzer (MPEA, Hansatech, UK) was used to record prompt chlorophyll fluorescence in the first 1 s of illumination with red light, and the PSII performance index (PI_abs_) was calculated according to Strasser et al. (2010).

### 2.7 Statistical analyses

Data are presented as the mean of samples from four replicate plots in the field trial. Means were tested for significant differences using an LSD test following one-way ANOVA. Statistical analyses were conducted in SPSS 22.0 (SPSS Inc., Chicago, IL, USA), and differences were considered significant at *P* < 0.05. Regression analysis was also performed using SPSS 22.0.

Structural equation modeling (SEM) was used to evaluate direct and indirect relations among N accumulation, C fixation, and yield in different fertilization treatments and was performed using AMOS Graphics 23.0 (IBM Corp., Armonk, NY, USA). The fit of the resulting model was evaluated using *P*-values, χ^2^ values, a goodness-of-fit index (GFI), and the root mean square error of approximation (RMSEA).

## 3 Results

### 3.1 Fruit yield, biomass, and nitrogen content and use efficiency

**Figure 1:**
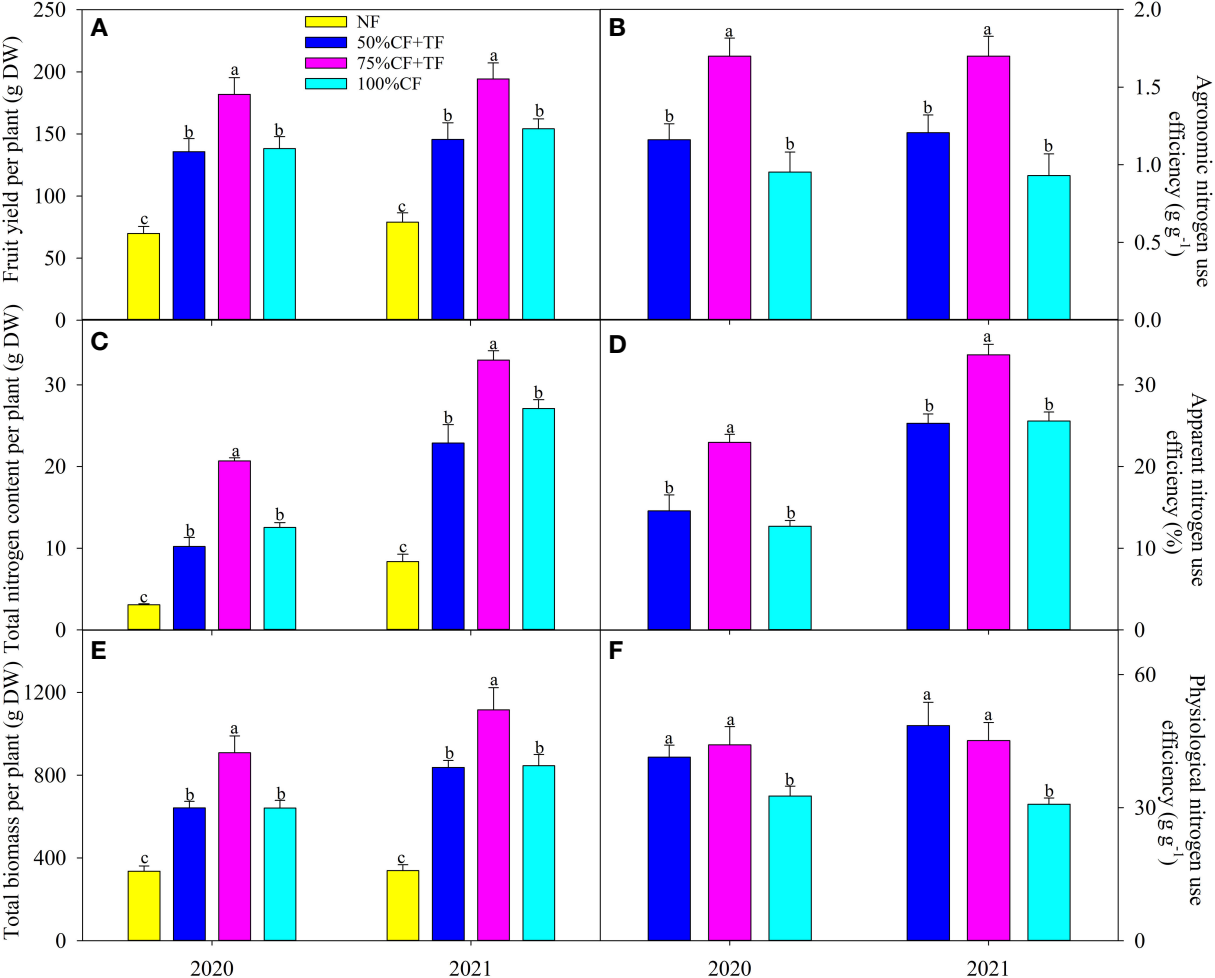
Wolfberry **(A)** fruit yield, **(C)** total nitrogen, and **(E)** biomass and **(B)** apparent, **(D)** physiological, and **(F)** agronomical nitrogen use efficiency in different fertilizer treatments in coastal saline land. Values are the mean of four replicate plots ( ± SD), and different letters indicate significant differences among treatments at *P* < 0.05. NF, no nitrogen fertilizer; 100% CF, 100% chemical nitrogen fertilizer; 50% CF+TF, 50% CF plus *Trichoderma* biofertilizer; 75% CF+TF, 75% CF plus *Trichoderma* biofertilizer.

Fruit yield, plant biomass, and N content increased significantly with application of N fertilizer, and the increase was significantly higher in plots with TF plus 75% CF than in plots with 100% CF and TF plus 50% CF (**Figures 1A, C, E**). Compared with 100% CF plots, agronomic, apparent, and physiological N efficiencies increased significantly in plots with TF plus 75% CF by 78.23%, 80.76%, and 35.32% in 2020 and by 82.70%, 31.67%, and 46.60% in 2021, respectively. However, compared with 100% CF, only physiological N efficiency increased significantly in plots with TF plus 50% CF (**Figures 1B, D, F**).

### 3.2 Leaf δ^13^C, δ^15^N, carbon, and nitrogen contents

**Figure 2:**
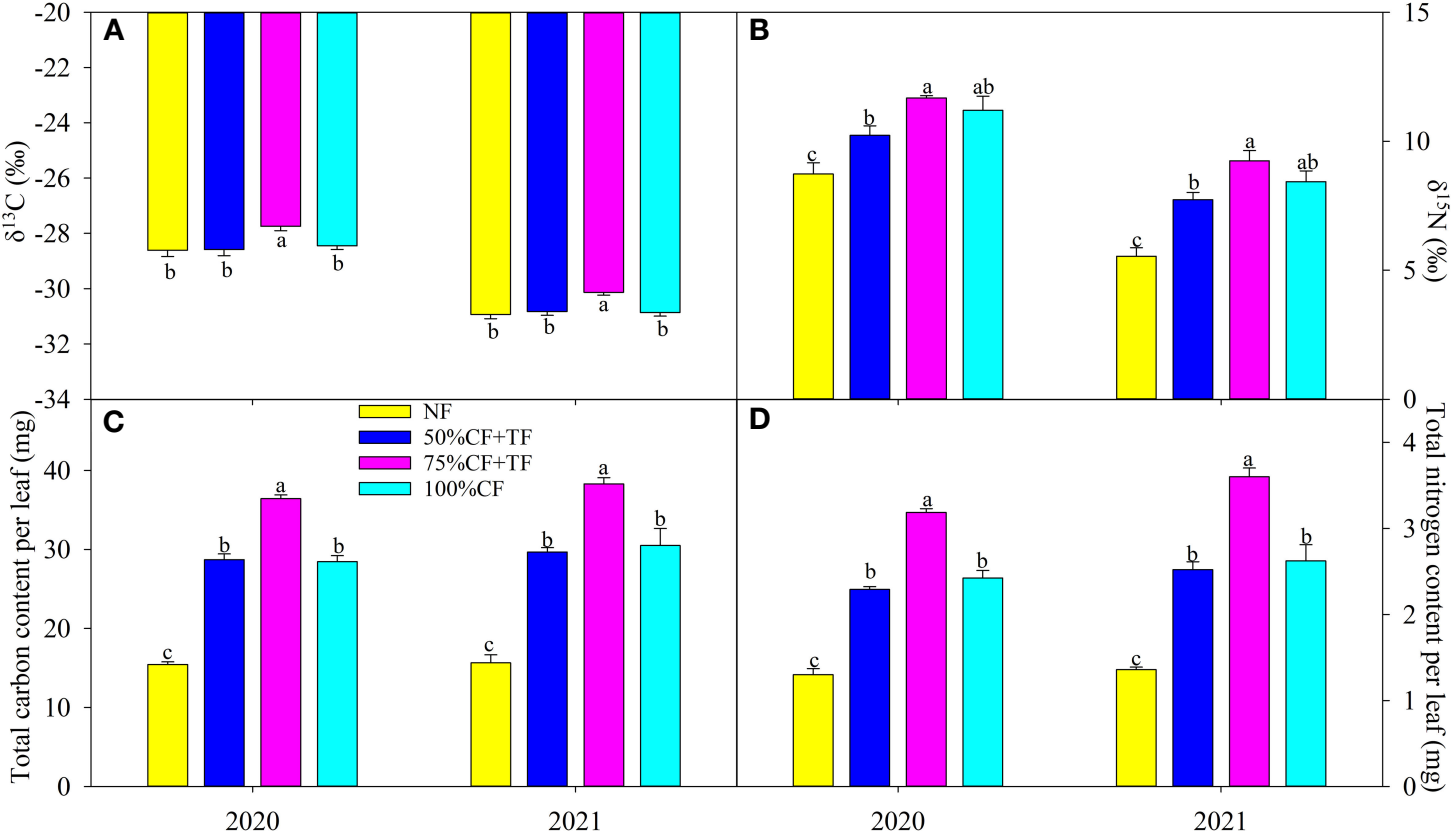
Leaf **(A)** carbon (δ^13^C) and **(B)** nitrogen (δ^15^N) isotope composition and **(C)** carbon, and **(D)** nitrogen accumulation in wolfberry in different fertilizer treatments in coastal saline land. Values are the mean of four replicate plots ( ± SD), and different letters indicate significant differences among treatments at *P* < 0.05. NF, no nitrogen fertilizer; 100% CF, 100% chemical nitrogen fertilizer; 50% CF+TF, 50% CF plus *Trichoderma* biofertilizer; 75% CF+TF, 75% CF plus *Trichoderma* biofertilizer.

Applying N fertilizer did not significantly affect leaf δ^13^C, except for a significant increase in TF plus 75% CF plots in 2020 and 2021 (**Figure 2A**). Leaf δ^15^N increased significantly with N fertilizer application, but there was no significant difference in leaf δ^15^N between plots with TF plus 75% CF and those with 100% CF (**Figure 2B**). Carbon and N contents per leaf increased significantly with application of N fertilizer, but the increases in TF plus 75% CF plots were significantly greater than those in 100% CF and TF plus 50% CF plots (**Figures 2C, D**).

### 3.3 Photosynthetic parameters in the field trial

**Figure 3:**
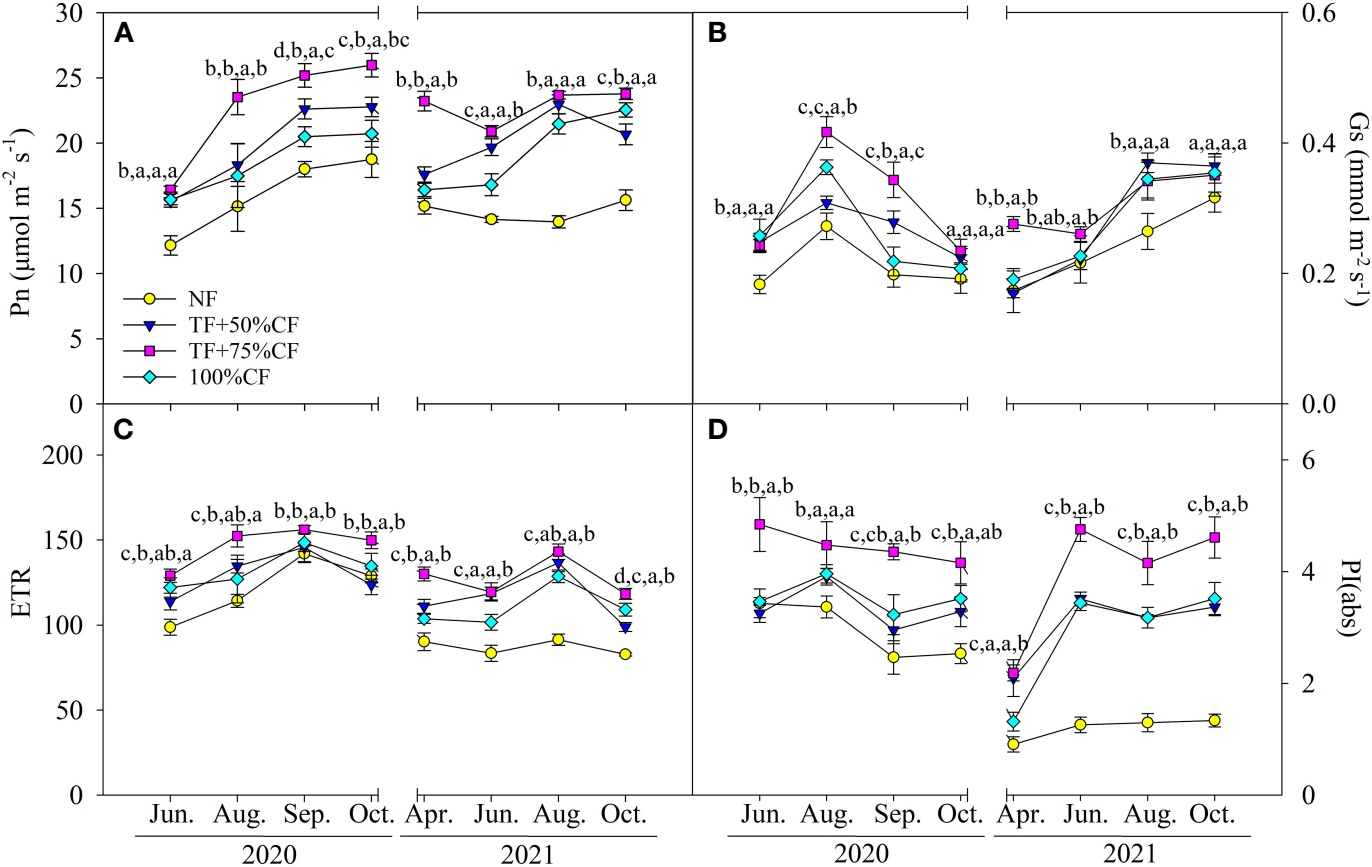
Wolfberry **(A)** photosynthetic rate (Pn), **(B)** stomatal conductance (Gs), **(C)** photosynthetic electron transport rate (ETR), and **(D)** performance index (PI_abs_) in different fertilizer treatments in coastal saline land. Values are the mean of four replicate plots ( ± SD), and different letters indicate significant differences among treatments at *P* < 0.05. NF, no nitrogen fertilizer; 100% CF, 100% chemical nitrogen fertilizer; 50% CF+TF, 50% CF plus *Trichoderma* biofertilizer; 75% CF+TF, 75% CF plus *Trichoderma* biofertilizer.

In 2020 and 2021 growing seasons, Pn, Gs, ETR, and PI_abs_ generally increased with N fertilizer application. Changes in those parameters were relatively greater in TF plus 75% CF plots than in 100% CF and TF plus 50% CF plots, although differences were not always significant in the monthly samplings (**Figure 3**).

### 3.4 Structural equation model analysis and relations between leaf nitrogen accumulation and photosynthetic rate, plant biomass, and fruit yield

**Figure 4:**
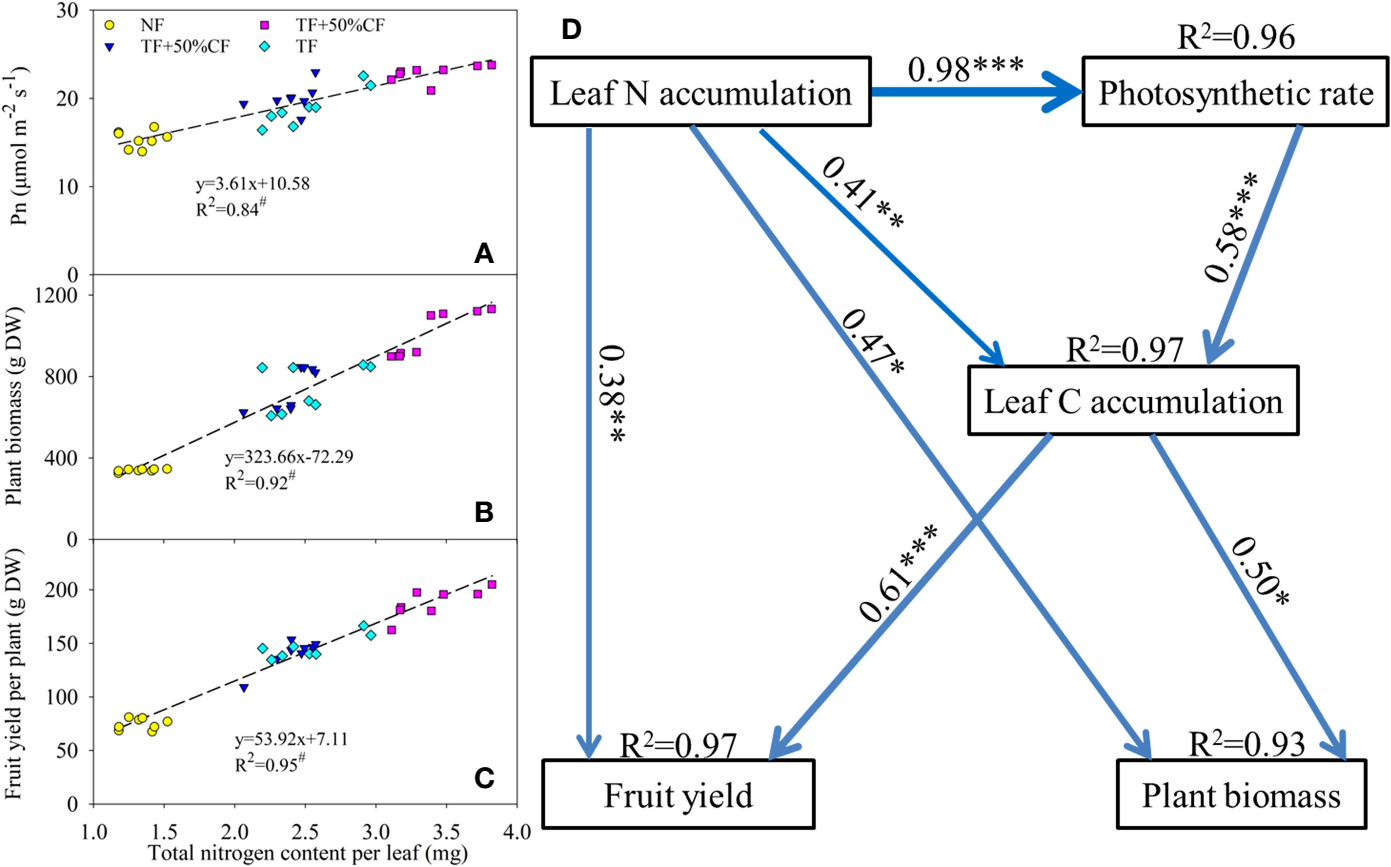
Regression analysis of leaf nitrogen accumulation with **(A)** photosynthetic rate (Pn), **(B)** biomass, and **(C)** fruit yield in wolfberry in coastal saline land. Significant relation at *P* < 0.01 is indicated by ^#^. NF, no nitrogen fertilizer; 100% CF, 100% chemical nitrogen fertilizer; 50% CF+TF, 50% CF plus *Trichoderma* biofertilizer; 75% CF+TF, 75% CF plus *Trichoderma* biofertilizer. **(D)** Structural equation model of hypothesized causal relations among leaf nitrogen and carbon accumulation, Pn, biomass, and yield formation in wolfberry in a two-year field trail. Model fit: χ^2 = ^0.415, *P* = 0.937, GFI = 0.995, RMSEA < 0.001. Blue solid lines indicate significant positive effects (*P* < 0.05). Standardized path coefficients are listed beside each path (line width indicates the proportion of factorial contribution). The *R* ^2^ values indicate the strength of explanation by independent variables. Significant effects: *P < 0.05; **P < 0.01; ***P < 0.001.

In regression analysis, leaf N accumulation was significantly positively related to Pn, plant biomass, and fruit yield (**Figures 4A-C**). An SEM analysis was conducted to identify direct and indirect relations among N accumulation, C fixation, plant biomass, and fruit yield (**Figure 4D**). In the SEM, leaf N accumulation directly positively regulated leaf C accumulation and Pn and also significantly indirectly affected fruit yield and plant biomass by affecting C fixation (**Figure 4D**). Thus, the coupling of accumulated N with C fixation was responsible for increases in plant growth and yield with TF substitution fertilization.

### 3.5 Soil total and mineral nitrogen contents

**Figure 5:**
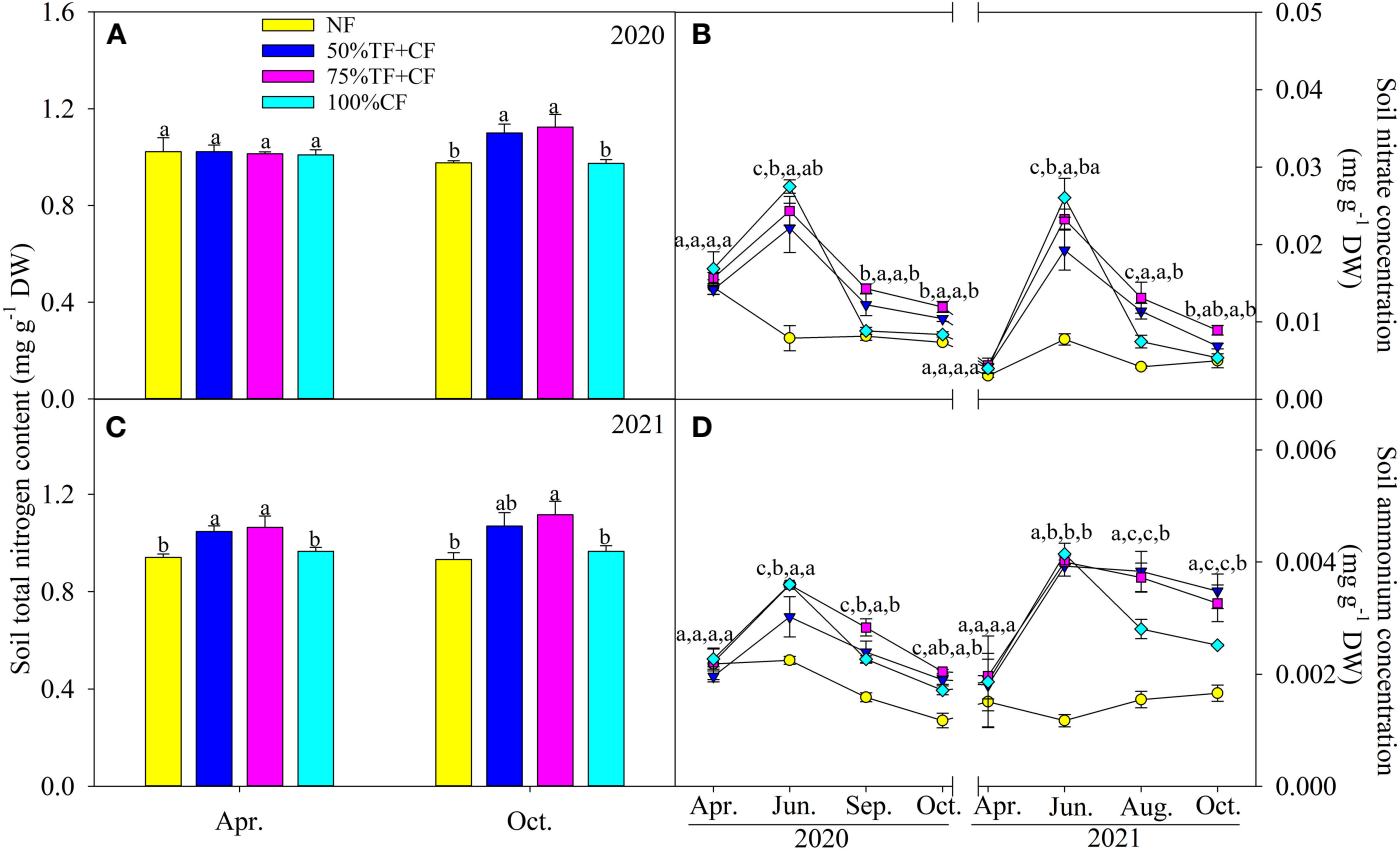
Topsoil total nitrogen content in **(A)** 2020 and **(C)** 2021 and **(B)** nitrate and **(D)** ammonium content in different fertilizer treatments in coastal saline land. Values are the mean of four replicate plots ( ± SD), and different letters indicate significant differences among treatments at *P* < 0.05. NF, no nitrogen fertilizer; 100% CF, 100% chemical nitrogen fertilizer; 50% CF+TF, 50% CF plus *Trichoderma* biofertilizer; 75% CF+TF, 75% CF plus *Trichoderma* biofertilizer.

Compared with NF and 100% CF plots, soil total N content increased significantly in TF plus 75% CF plots at the end of the growth seasons in October 2020 and 2021 (**Figures 5A, C**). Soil NH_4_^+^-N and NO_3_^–^N contents increased significantly in June in plots with N fertilizer application and then gradually declined during the growing seasons (**Figures 5B, D**). The decreases in soil NH_4_^+^-N and NO_3_^–^N contents after June were not as great in TF plus 75% CF plots as in 100% CF plots, and soil NH_4_^+^-N and NO_3_^–^N contents remained generally higher in TF plus 75% CF plots in the second half of the seasons (**Figures 5B, D**).

## 4 Discussion

High N application is commonly used for planting wolfberry by farmers because N in saline soil is liable to loss, and maybe, a lower chemical N application rate is appropriate considering that excessive N availability can lead to a systemic repression of root growth with yield reduction (Giehl and von Wiren, 2014; Liu et al., 2022). For example, Liu et al. (2018) reported that high N application rate at 300 kg ha^-1^ reduced wheat yield and root weight density in contrast to an optimal application rate at 240 kg ha^-1^. However, this study focused on the approach of TF substitution to reduce chemical N application. TF substitution could reduce 50% CF investment without influencing wolfberry growth and yield in contrast to 100% CF application, and notably, TF substitution with 25% reduction of chemical fertilizer improved wolfberry growth and yield (**Figures 1A, E**). Thus, TF proved to be functional and beneficial for wolfberry cultivation in coastal saline land. The growth-promoting effect of TF probably related to N uptake and use, considering greater plant nitrogen accumulation in plots with TF plus 75% CF than 100% CF (**Figure 1C**). Consistently, higher apparent, physiological, and agronomic N use efficiencies in plants with TF plus 75% CF further verified that growth-promoting function of TF involved plant N uptake and use (**Figures 1B, D, F**). As a proxy of N uptake and use in plants, δ^15^N has a positive relation with crop growth and yield (Yousfi et al., 2009; Yousfi et al., 2012). In this study, δ^15^N increased in plants with N fertilization. However, the relatively minor difference in leaf δ^15^N did not match the greater yield and biomass in 75% CF plus TF plots than in 100% CF plots (**Figures 1A, C**; **2B**). Nitrogen isotope fractionation mainly occurs during enzymatic assimilation of nitrate or ammonium into organic forms. High N assimilation capacity can directly increase δ^15^N by decreasing discrimination against ^15^N, whereas high N uptake indirectly increases discrimination against ^15^N by increasing available N (Evans, 2001; Tcherkez, 2011). Thus, greater N uptake in plants with TF plus 75% CF than with 100% CF might attenuate leaf ^15^N discrimination. Yousfi et al. (2012) also found increases in biomass and N accumulation with insignificant variation in shoot δ^15^N in salt-tolerant cultivar RIL47 of durum wheat in contrast to salt-sensitive cultivar RIL 24 under salt stress. Overall, TF substitution increased wolfberry N uptake and use in coastal saline land.

Similar to the accumulation of plant N, the stock of soil total N also increased in 75% CF plus TF plots compared with 100% CF plots at the end of growth seasons (**Figures 5A, C**), suggesting that TF increased retention of fertilizer N in saline soil and as a result, could benefit plant N uptake. This finding was supported by previous reports that organic supplements could inhibit N loss through NH_3_ volatilization from saline soil (Al-Busaidi et al., 2014; Xiao et al., 2020; Yao et al., 2021). Notably, in a pot experiment, N storage in saline soil increased with application of *Trichoderma* spore powder (Daliakopoulosa et al., 2019). In addition, in contrast to 100% CF plots, TF substitution mitigated the steep decrease in soil mineral N concentrations that occurred in the second half of growing seasons. Therefore, by slowing the decline in soil mineral N concentrations, TF increased stability of soil mineral N supply (**Figures 5B, D**). In agreement with our finding, Wang et al. (2018) found that in a pot experiment, TF reduced NH_3_ volatilization loss from saline soil by increasing soil nitrification which also increased N supply. Recent studies also show that addition of organic materials such as crop straw can optimize soil N supply by regulating N transformations (Lu et al., 2018; Li et al., 2020a; Chen et al., 2021; Yuan et al., 2021). Therefore, soil N transformations in coastal saline land under combined application of CF with TF should be investigated further. However, in the field trial in this study, TF substitution helped to improve soil N supply to plants in coastal saline land.

Plant N use for growth and yield formation is dependent on photosynthetic C fixation. The greater increase in Pn with 75% CF plus TF than with 100% CF suggested that TF substitution helped increase photosynthetic capacity in wolfberry (**Figure 3A**). PSII is the initiation site for driving photosynthetic electron transport, and it is also susceptible to photoinhibition under environmental stresses (Takahashi and Murata, 2008). In accordance with Pn, the increase in PI(abs) was greater with 75% CF plus TF than with 100% CF, which maintained ETR at a higher level to promote C assimilation (**Figures 3C, D**). In addition, the greater increase in Gs with 75% CF plus TF than with 100% CF also benefited photosynthesis by increasing CO_2_ diffusion into leaves (**Figure 3B**). Increases in CO_2_ supply with high Gs tend to decrease δ^13^C by increasing ^13^C fractionation, whereas strong CO_2_ fixation can depress discrimination against ^13^C and increase δ^13^C (Farquhar et al., 1989; Li et al., 2017). Thus, the increase in δ^13^C without reduction in Gs indicated greater CO_2_ fixation in plants with 75% CF plus TF than with 100% CF (**Figures 2A**; **3B**). Overall, TF substitution improved photosynthetic capacity of wolfberry in coastal saline land and then increased leaf C accumulation (**Figure 2C**). Importantly, leaf N accumulation was significantly and positively related with Pn, biomass, and fruit yield, and the SEM also confirmed the importance of the causal relation of N accumulation coupled with C fixation for biomass and yield formation (**Figure 4**). Therefore, elevated N accumulation could well couple with photosynthetic C fixation to increase wolfberry biomass and yield with 75% CF plus TF, and led to higher physiological and agronomic nitrogen use efficiency (**Figures 1B, F**).

## 5 Conclusion

In summary, partial substitution of CF by TF improved N use efficiency in wolfberry in coastal saline land by stabilizing soil N supply and increasing the synergism between N accumulation and C fixation.

## Data availability statement

The raw data supporting the conclusions of this article will be made available by the authors, without undue reservation.

## Author contributions

KY designed the experiment, performed the experiment, and wrote the manuscript. YR and HS participated in data analysis. HM, SY, YZ, SL, and YS participated in the experiment. All authors contributed to the article and approved the submitted version.
